# When Metrics Replace Values: Ethical Concerns in Healthcare Performance Measures

**DOI:** 10.1111/acem.70330

**Published:** 2026-05-18

**Authors:** Neeraj Chhabra

**Affiliations:** ^1^ Department of Emergency Medicine University of Illinois Chicago Chicago Illinois USA; ^2^ MacLean Center for Clinical Medical Ethics University of Chicago Chicago Illinois USA

Healthcare is increasingly reliant on quantitative metrics to evaluate quality and promote accountability [1]. These measures are intended to improve patient outcomes by promoting best practices and reducing variability in care [2]. Goodhart's Law warns that “when a measure becomes a target, it ceases to be a good measure” [3]. This axiom highlights a growing ethical tension in medicine: Metrics designed as tools for improving patient care often become ends in themselves, distorting priorities and undermining core values. The emergency department (ED) metric, Left Without Being Seen (LWBS), illustrates how distortion occurs and raises concerns about distributive justice, beneficence, and professional integrity.

LWBS refers to the proportion of patients that leave an ED prior to clinician evaluation. It emerged as a proxy for emergency care quality, hospital crowding, and access to medical care. Increased LWBS is associated with adverse outcomes making it an attractive metric to quantify access to timely acute care [4]. Policymakers and administrators, including those at the Centers for Medicare and Medicaid Services, incorporated LWBS into quality reporting frameworks, reinforcing its status as a benchmark of hospital performance [2].

As LWBS became linked to reimbursement and institutional reputation through public reporting, it evolved from a metric into a performance target [5, 6]. This shift has altered behavior. Organizations are incentivized to focus on reducing the LWBS percentage in the ED, rather than improving patient access to healthcare more broadly. This shift has invited “gaming,” where strategies are employed to explicitly improve measured performance without attention to the material improvement of underlying system conditions.

Although poor LWBS performance indicates broader health system concerns, EDs have been primarily tasked with improving the metric as its site of measurement. EDs have responded to LWBS pressures with workflow adaptations that prioritize metric compliance. Common strategies include placing providers in waiting rooms or triage areas to ensure patients are technically “seen” while conducting brief evaluations. Other interventions have prioritized rapid evaluation of more numerous low acuity patients over “sicker” high‐acuity ones [7]. While such interventions achieve their primary goal, a reduction in ED LWBS proportion, they do so while shifting priorities from expanding hospital capacity, increasing access to timely outpatient care, and improving patient outcomes. This creates a value conflict between performance metrics and the intrinsic goals of medicine. As previous studies have shown, processes that improve the LWBS metric do not always translate to material improvement of patient outcomes, raising concerns about beneficence [8, 9]. Systemic constraints, and not merely front‐end processes, drive patient outcomes. Narrowly focusing on improving LWBS masks deeper structural problems.

The benefits of LWBS interventions must be weighed against resource costs and tradeoffs in care prioritization. EDs increasingly operate under conditions of scarcity, where clinician time and physical space are limited. Allocating additional clinician time to triage roles diverts resources away from other critical tasks. Patients with LWBS are disproportionately represented by younger and lower acuity patients [10, 11]. Prioritization of rapid evaluation for low acuity patients over caring for patients with high acuity illnesses undermines the core purpose of emergency care settings [7, 12]. Through the lens of distributive justice, this represents a misallocation of scarce resources.

The impacts of metric “gaming” extend to both patients and clinicians. Patients may receive rushed evaluations to fulfill metric requirements without having their primary concerns adequately addressed. Vulnerable patients are disproportionately represented among patients with LWBS and may continue to experience unmet medical needs despite the superficial appearance of improved care [13]. Provider‐in‐triage models establish a physician–patient relationship, the duties of which ED clinicians may not be able to meet. The duty to provide competent care, components of which are continued care or appropriate transition of responsibility to a colleague, may not be met by the provider‐in‐triage model as most patients return to a waiting area following initial evaluation without explicit care transition [12]. Clinicians may experience moral distress when institutional pressures compel them to prioritize metrics over their fiduciary duties to patients. It is unsurprising that metric‐driven environments erode clinicians' sense of integrity and agency [14, 15].

Despite these concerns, the need to measure care quality in medicine remains. Metrics can identify system constraints and, even when imperfect, can drive meaningful innovation and improvement. They are also essential for benchmarking and accountability. Gaming can represent adaptive workflow changes rather than overt ethical failure, and such change can still achieve a dual purpose which includes clinical benefit. Yet a tension persists; metrics can catalyze improvement while distorting priorities away from core medical values.

Overreliance on single and proxy metrics oversimplifies the complexities of healthcare delivery [1, 16]. When “gaming” occurs, it is a signal that incentives may be misaligned from goals of medical care. Widely adopted quality measures result from years of research, stakeholder consensus, validation, and resource‐intensive implementation creating inertia that makes revisions or abandonment difficult. This reflects a sunk cost dynamic where prior investment sustains reliance on measures that no longer serve their purpose. To combat this, metrics tied to reimbursement should be designed as provisional tools with defined lifecycles that include periodic reevaluation and the capacity for revision or straightforward retirement as evidence of unintended consequences emerge. Embedding flexibility through patient outcome‐centric metric bundles, rotating incentives, and formal reassessments can preserve their value while preventing entrenchment [17].

The case of LWBS illustrates a broader ethical problem: metrics are not neutral. When they become targets, they reshape behavior in ways that can conflict with the fundamental aims of medical practice. LWBS demonstrates how well‐intentioned measures can undermine the values they seek to promote. We argue for a flexible approach to quality measurement that prioritizes ethical principles, resists distortions, and protects physicians' duties to patients.

## Conflicts of Interest

The author declares no conflicts of interest.

## Data Availability

Data sharing not applicable to this article as no datasets were generated or analysed during the current study.

## References

[1] R. J. Panzer , R. S. Gitomer , W. H. Greene , P. R. Webster , K. R. Landry , and C. A. Riccobono , “Increasing Demands for Quality Measurement,” JAMA 310, no. 18 (2013): 1971–1980. 24219953 10.1001/jama.2013.282047

[2] S. Das , G. Miller , C. Libby , C. J. Gettel , E. Rabin , and M. Lin , “Why Do We Need Quality Measures in Emergency Medicine?,” Journal of the American College of Emergency Physicians Open 5, no. 6 (2024): e13329. 39697807 10.1002/emp2.13329 PMC11652395

[3] C. Mattson , R. L. Bushardt , and A. R. Artino , “When a Measure Becomes a Target, It Ceases to be a Good Measure,” Journal of Graduate Medical Education 13, no. 1 (2021): 2–5. 33680290 10.4300/JGME-D-20-01492.1 PMC7901608

[4] C. D. McNaughton , P. C. Austin , A. Chu , et al., “Turbulence in the System: Higher Rates of Left‐Without‐Being‐Seen Emergency Department Visits and Associations With Increased Risks of Adverse Patient Outcomes Since 2020,” JACEP Open 5, no. 6 (2024): e13299. 39703807 10.1002/emp2.13299 PMC11655912

[5] S. R. Wing , C. Barclay‐Buchanan , S. Arneson , et al., “Reduced Left‐Without‐Being‐Seen Rates and Impact on Disparities After Guest Services Ambassadors Implementation,” Academic Emergency Medicine: Official Journal of the Society for Academic Emergency Medicine 32, no. 3 (2025): 216–225. 39910715 10.1111/acem.15100 PMC11921064

[6] M. Sember , C. Donley , and M. Eggleston , “Implementation of a Provider in Triage and Its Effect on Left Without Being Seen Rate at a Community Trauma Center,” Open Access Emerg Med OAEM 13 (2021): 137–141. 33824606 10.2147/OAEM.S296001 PMC8018550

[7] G. Jasani , Revisiting “Left Without Being Seen” Metrics in the ED [Internet] (2024), cited Apr 9, 2026, https://www.medpagetoday.com/opinion/second‐opinions/110212.

[8] J. D. Schuur , R. Y. Hsia , H. Burstin , M. J. Schull , and J. M. Pines , “Quality Measurement in the Emergency Department: Past and Future,” Health Affairs 32, no. 12 (2013): 2129–2138. 24301396 10.1377/hlthaff.2013.0730

[9] A. U. Joshi , F. T. Randolph , A. M. Chang , et al., “Impact of Emergency Department Tele‐Intake on Left Without Being Seen and Throughput Metrics,” Academic Emergency Medicine: Official Journal of the Society for Academic Emergency Medicine 27, no. 2 (2020): 139–147. 31733003 10.1111/acem.13890

[10] D. R. Li , J. J. Brennan , A. A. Kreshak , E. M. Castillo , and G. M. Vilke , “Patients Who Leave the Emergency Department Without Being Seen and Their Follow‐Up Behavior: A Retrospective Descriptive Analysis,” Journal of Emergency Medicine 57, no. 1 (2019): 106–113. 31078346 10.1016/j.jemermed.2019.03.051

[11] N. K. Rathlev , P. Visintainer , J. Schmidt , J. Hettler , V. Albert , and H. Li , “Patient Characteristics and Clinical Process Predictors of Patients Leaving Without Being Seen From the Emergency Department,” Western Journal of Emergency Medicine 21, no. 5 (2020): 1218–1226. 32970578 10.5811/westjem.2020.6.47084 PMC7514399

[12] Code of Ethics for Emergency Physicians , “Code of Ethics for Emergency Physicians,” Annals of Emergency Medicine 70, no. 1 (2017): e7–e15. 28645421 10.1016/j.annemergmed.2017.03.032

[13] M. Schick , N. Fumo , L. Nickel , J. Aranda , R. Mackenzie , and N. Jacobson , “Social Vulnerability Index Predictive of Being “Left Without Being Seen” in the Emergency Department,” American Journal of Emergency Medicine 96 (2025): 191–196. 40580883 10.1016/j.ajem.2025.06.032

[14] M. F. MacIsaac , L. Pollack , G. Massaro , L. Marshall , G. Crema , and D. Brener , “Reimagining Quality Metrics: A Physician‐Centered Approach to Healthcare Improvement,” American Journal of Medicine 137, no. 11 (2024): 1059–1062. 39094844 10.1016/j.amjmed.2024.07.012

[15] R. Winslow , “Failing the Metric but Saving Lives: The Protocolization of Sepsis Treatment Through Quality Measurement,” Social Science & Medicine 253 (2020): 112982. 32298917 10.1016/j.socscimed.2020.112982

[16] M. P. A. JLB , Overhauling Quality Measurement in the US: Measure What Matters | AJMC [Internet] (2026), cited Apr 9, 2026, https://www.ajmc.com/view/overhauling‐quality‐measurement‐in‐the‐us‐measure‐what‐matters.

[17] D. Manheim , “Building Less‐Flawed Metrics: Understanding and Creating Better Measurement and Incentive Systems,” Patterns 4, no. 10 (2023): 100842. 37876897 10.1016/j.patter.2023.100842 PMC10591122

